# Progress in Medicine

**Published:** 1899-02-04

**Authors:** 


### Progress in Medicine.

### THE CIRCULATORY SYSTEM.

(*Continued from page* 279.)

**Prognosis.**—Prognosis in cases of cardiac diseases in its bearings upon life assurance was discussed at the annual meeting of the British Medical Association.[5] Gairdner pointed out that our present knowledge was insufficient for reliable information on this subject, and that help was to be looked for from the records of private practice. He instanced two cases in which mitral murmurs after being present for over thirty years had disappeared, and a third in which the patient survived for ten years, although the tricuspid orifice was obstructed by a tumour. The only alternative to "loading" or rejecting a life was to postpone the examination as long as possible, keeping the applicant under observation. The indefinite murmurs sometimes heard at the base of the heart he regarded as exocardial. Acute pericarditis does not necessarily lead to adhesion, and may leave behind it no recognisable signs and symptoms. Organic lesions without functional disturbances are not rare. Such cases should be watched before the life is either loaded or rejected. Senile degeneration should either lead to absolute rejection or a heavy addition of years. Persons with complicated organic mischief are uninsurable. Functional disorders are difficult to deal with, intermittency and irregularity being sometimes compatible with apparent health. Bradycardia is of more significance than tachycardia, but a moderately slow pulse of 60 is consistent with health. Choreic heart murmurs are usually organic. An abnormal enlargement is probably a factor of more grave importance than a small heart. A difficulty arose in distinguishing between angina and pseudo-angina. Theodore Williams[6] thinks that persons suffering from adherent pericardium should be accepted with an addition of three to five years, provided there be no dilatation or valvular lesion. Similarly, uncomplicated cases of mitral regurgitation, if compensation be good, will call for an addition of five or ten years. Mitral stenosis cases are less favourable, Aortic valvular disease will generally exclude from insurance. Cardiac dilatation should be watched, as it may be only of a temporary nature. Cases of cardiac hypertrophy will generally call for the addition of an extra number of years, according to the age of the patient. All forms of cardiac neurosis and degeneration should be excluded.

**Treatment.**—Jacobœus[7] advises that heart failure be first treated by rest alone, resort being made afterwards to strophanthus and then to digitalis. Tincture of strophanthus is the best preparation. It acts more rapidly than digitalis, is not cumulative in its action, and has no effect on the peripheral vessels. Digitalis may be harmful in many forms of cardiac disease, as by acting upon the vaso-motor constrictors it raises the blood pressure. The therapeutic activity of different preparations of digitalis varies very considerably. It has been questioned whether the fluid preparations are active when manufactured into tablets, but Houghton,[7] as the result of a number of experiments conducted on animals, finds that there is no loss of activity even if the tablets be kept for a prolonged period of time. Of the commercial non-official preparations of digitalis, crystalised digitoxin is probably the most influential for good,[8] being preferable to German or French digitalin. The dose is ·00025 gme., three or four times a day. Its action is markedly slow, and it is cumulative.

Barium chloride resembles digitalis in its action.[9] It increases blood pressure and slows the pulse by prolonging and strengthening systole. It is especially suitable for cases of failing compensation, both in adults and children, especially when the pulse is weak, irregular, and lacking in volume. The dose for an adult is a teaspoonful of the 1 per cent. solution three times daily. Poulet[10] advocates the use of an extract or infusion of the leaves and flowers of coronilla varia. The action is that of digitalis or strophanthus, but the drug has the advantages of being non-cumulative, and of exercising a tonic effect upon the digestive system. Its diuretic action is small. It is especially useful in arhythmia and tachycardia, in cardiac giddiness, and in cases where the digestive functions are disturbed.

[5] Brit. Med. Jour., Sept. 17, 1898. [6] Med. Examiner, July, 1898. [7] Klinisch-therapeutische Wochenschrift, 11-14, 1898. [8] Amer. Med. and Surg. Bull., July 25, 1898. [9] Therapeutic Gazette, April 15, 1898. [10] Les Nouveaux Remèdes, July, 1898.

### NEUROLOGY.

**The Neurone Theory of the Nervous System** has recently been discussed by Barker[1] in an interesting article. The theory was first formulated by Waldeyer about seven years ago, though much of the work on which it was based had been done years before. The theory is that the nervous system consists of numerous nerve units (neurones) united to one another neither anatomically nor genetically. Every nerve unit is composed of three pieces, the nerve cell, the nerve fibre, and the terminal arborescence. The doctrine was founded on the following data: (1) The *à priori* probability that the nervous system, like other parts of the body, is a cellular system; (2) the proof that in the embryo the nerve cells exist as independent units, many of which are capable of wandering to a considerable distance from their point of origin; (3) the proof that the nutrition of the nerve cells is most easily explicable from the standpoint of the doctrine which looks upon the nervous system as made up of units, since in pathological degenerative processes affecting a unit or a set of units degeneration of a given type extends only within the limits of that unit or set of units; and (4) the histological demonstration of the fact that sometimes one unit, sometimes another, may be picked out by a particular method of staining, the others remaining only partially stained (*e.g.*, by Golgi's process). Observations made since 1891, the date of Waldeyer's article, have in the main tended to support the doctrine. The investigations of degenerations by Marchi's method, by which degeneration can be detected at an earlier date than is possible by Weigert's stain, confirm the results of the latter method, that degeneration following an injury does not extend beyond the neurones involved in the injury. If degeneration in one set of neurones spreads to another, as it may do after a very long time, the change in the second set is one of shrinking and diminution of calibre of the medullated fibres rather than complete disintegration and absorption. Again, investigators who have employed Nissl's method have discovered facts which harmonise with the neurone doctrine. For instance, when any portion of the axis-cylinder process of a nerve cell is diseased, the whole neurone to which that process belongs suffers; thus changes take place in the "stainable substance" of the cell body and the other cell processes of that nerve cell.

Held agrees with other investigators that in embryonic tissues and early youth the neurones are entirely independent of one another. In these stages he finds, when the terminal of an axis cylinder process comes into contact with the cell body of another neurone, he can always make out where the protoplasm of one neurone begins and another ends, insomuch as the line of demarcation is more refractive than the adjacent protoplasm. In adult life, however, this line disappears, so that he comes to the conclusion that the protoplasm of related neurones fuses. It is, however, possible to distinguish between the two cells by the distribution of the small particles in their protoplasmic groundwork. Held's discoveries are thus confirmatory of the neurone doctrine.

Apáthy's investigations at Naples on the nervous system of the earthworm and leech have by some been thought to be subversive of the neurone doctrine, but this is very doubtful. Many of the apparent findings, *e.g.*, the unipolarity of the nerve cells, the presence of fibrils in the processes and reticula of the cell body, are really old, though having the appearance of novelty owing to the absence of discussion of previous men's work. And even if his doctrines are accepted, which seems doubtful, it would not follow that the neurone doctrine of Waldeyer would have to be abandoned. Barker comes to the conclusion that, as formulated by Waldeyer, the human nervous system is made up largely of great numbers of cell-units, the so-called neurones, the existence of which is too firmly established ever to be utterly overthrown.

**General Paralysis of the Insane.**—Sachs[2] discusses the difficulties which beset the diagnosis of paretic dementia in its early stages. The fully-fledged form of the disease bears its unmistakeable symptoms, but these are variable and poorly defined in the onset. The physical signs often, though not invariably, precede the mental; but a diagnosis should not be ventured on unless there be at least some indications of a change in the person's psychic state. The more important of these physical signs are: (1) stammering, tremulous speech; (2) tremor of the facial muscles and of the tongue; (3) the pupillary symptoms; (4) the change in the individual's handwriting; (5) the exaggeration or absence of the reflexes.

In regard to these signs it is to be remarked that disturbance of speech is one of the earliest symptoms, and one is frequently led to make a diagnosis from this if there be also some mental change. But mental deterioration of alcoholic origin may cause an undistinguishable stammering utterance. Similarly chronic alcoholism may bring about tremor of the facial muscles. If alcohol can be excluded, these two physical signs are of the highest significance. The pupillary symptoms may in exceptional cases not be developed until after the characteristic speech disturbances and facial tremor. The typical Argyll-Robertson pupil is common, particularly in those forms associated with tabetic symptoms; also complete immobility of the pupils, which are often unequal. The changes in the handwriting may illustrate not only the tremor of the fingers but also the beginning of mental dissolution; thus, there may be dropping of letters from words which were previously written with ease, or the omission of syllables, or the running together of words which should be separated. Too much importance should not be attached to the tremor alone, as it may occur in other diseases. If the reflexes be absent they may be part of an accompanying tabetic process; whilst, if exaggerated, a purely neurasthenic condition must be excluded before any weight can be given to the phenomena in support of the diagnosis of general paresis.

The normal individual near or past middle life is so much the creature of well-established habits of thought and action, that any departure from the standard he has set himself should be viewed with considerable suspicion, *e.g.*, unaccustomed extravagances or economies, indifference to family ties, defective judgment, defective memory.

The early stages of general paralysis may be confounded with neurasthenia. In that complaint, however, as a rule, the complaints are of a hypochondriacal order, the loss of memory is more apparent than real, and in writing, though the hand may tremble, yet letters and syllables are not omitted. In cases where some doubt remains it should be remembered that the neurasthenic recovers rapidly under the influence of rest, whilst though the paretic may quiet down a little the physical symptoms once established do not disappear. In some cases general paralysis can be with difficulty distinguished from alcoholic dementia, and sometimes the diagnosis is impossible for a time. If such symptoms as marked tremor of the tongue and hands, paræsthesia, and signs of peripheral neuritis are present in a case exhibiting some symptoms of dementia paralytica, the diagnosis of the latter disease should be held in abeyance for a considerable length of time.

[1] Barker, Amer. Jour. of Insanity, July, 1898. [2] Sachs, New York Med. Jour., July 2 and 9, 1898.

